# Supplementary material for: Exploration of the Regulatory Pathways and Key Genes Involved in the Response to Saline–Alkali Stress in Betula platyphylla via RNA-Seq Analysis
Source: Plants (Basel). 2023 Jun 24;12(13):2435. doi: 10.3390/plants12132435 (PMC10346881; doi:10.3390/plants12132435)
Supplement: Supplementary file 1 [file plants-12-02435-s001.zip › Table.pdf]

Table S1: RNA-seq data statistics

Names		GC Content	%≥Q30
	ck-1	46.6%	93.95%
Control	ck-2	46.65%	93.68%
	ck-3	46.43%	93.6%
	N3-1	46.3%	93.5%
NaHCO3 3 h	N3-2	46.49%	92.83%
	N3-3	46.45%	93.56%
	N6-1	46.44%	92.8%
NaHCO3 6 h	N6-2	46.43%	93.01%
	N6-3	46.45%	93.54%

Table S2 The alignment statistics of the sequencing data against the reference genome

Names		Total Reads	Mapped Reads	Uniq Mapped Reads	Multiple Map Reads	Reads Map to +'	Reads Map to '-'
Control	ck-1	45,882,766	42,340,738 (92.28%)	41,306,864 (90.03%)	1,033,874 (2.25%)	21,823,009 (47.56%)	21,857,358 (47.64%)
	ck-2	44,678,562	41,588,698 (93.08%)	40,602,072 (90.88%)	986,626 (2.21%)	21,400,152 (47.90%)	21,439,696 (47.99%)
	ck-3	43,845,858	40,467,813 (92.30%)	39,574,236 (90.26%)	893,577 (2.04%)	20,796,645 (47.43%)	20,817,791 (47.48%)
$NaHCO_3$ 3 h	N3-1	54,155,330	50,068,330 (92.45%)	48,924,515 (90.34%)	1,143,815 (2.11%)	25,747,176 (47.54%)	25,781,187 (47.61%)
	N3-2	41,592,742	38,457,038 (92.46%)	37,573,043 (90.34%)	883,995 (2.13%)	19,779,094 (47.55%)	19,804,249 (47.61%)
	N3-3	59,572,294	55,228,967 (92.71%)	53,974,047 (90.60%)	1,254,920 (2.11%)	28,412,360 (47.69%)	28,437,763 (47.74%)
$NaHCO_3$ 6 h	N6-1	52,658,328	48,754,408 (92.59%)	47,637,470 (90.47%)	1,116,938 (2.12%)	25,066,454 (47.60%)	25,105,916 (47.68%)
	N6-2	49,886,458	46,068,439 (92.35%)	44,949,505 (90.10%)	1,118,934 (2.24%)	23,731,050 (47.57%)	23,772,214 (47.65%)
	N6-3	51,890,900	47,989,877 (92.48%)	46,877,643 (90.34%)	1,112,234 (2.14%)	24,691,964 (47.58%)	24,724,644 (47.65%)

Table S4: The primer sequences used for qRT-PCR analyses

Primer	Sequences(5'-3')	Amplicon length (bp)
Ubiquitin-F	GGAGGACAAGGTGGAGGG	228
Ubiquitin-R	GATTGAGGGGAGGGATGC	
α-tubulin-F	TGGCTCGAATGCACTGTTGG	215
α-tubulin-R	TCAACCGCCTTGTCTCTCAGG	
CYP82C2-F	GTGGAACAGGGACAT	159
CYP82C2-R	TTGGAAGAAAGAGCC	
WRKY70-F	CGATGATGTCACCCAC	157
WRKY70-R	ATCGGCAAAGTGGTC	
NAC9-F	TTTGTGCCGAGTGTA	176
NAC9-R	CTCCCGAGTAAGAAGT	
RLP53-F	GAAGGCTGAACACGC	180
RLP53-R	TGAAGCTGCTCCAAA	
ATJ11-F	CCATTCAGCGTATTCA	159

ATJ11-R	TCAGTCTCCCAGTTCC	
GSTF2-F	CACATACCTGCCTTCG	160
GSTF2-R	GTGCCTCCACTTCCAT	
XTH6-F	GCGATAGGGAACAAA	161
XTH6-R	CATCTTCGGGTAGGG	
XTH15-F	GGGAAATCAAAGGAA	158
XTH15-R	TTGGGCATCTGTAGTT	
PMEI7-F	AACCAGCTCAGCCAGTC	178
PMEI7-R	TCGCCTTCAACTTTCC	
WRKY41-F	AGATGCCATATCCTTT	168
WRKY41-R	CTTGGAAGTTGCTCGT	
BG2-F	GTGGAACAGGGACAT	177
BG2-R	TTGGAAGAAAGAGCC	
EARLI1-F	CTTCAACTTATGTCCCTT	187
EARLI1-R	GGTTTCTTTGGTGGG	

Table S5 Primers used for the construction of birch plants overexpressing *WRKY70* and *NAC9*

Primer	Sequences(5'-3')	Amplicon length (bp)
WRKY70-Infusion-F	GACTCTAGAGGATCCCCGGGATGGAGTCTTCTTGGCC	924
WRKY70-Infusion-R	ATTCGAGCTCGGTACCCGGGTTAAAAAGAAAAGGCACCC	
NAC9-Infusion-F	GACTCTAGAGGATCCCCGGGATGGAGGAATTCGCACC	744
NAC9-Infusion-R	ATTCGAGCTCGGTACCCGGGTTAAAGCCAATTCAGTTGT	

Table S6 Primer sequences of *SOD* and *POD* used in qRT-PCR analyses

Primer	Sequences(5'-3')	Amplicon length (bp)
SOD1-F	CCGTGGTTGTTCTTGGCAAC	190
SOD1-R	CCAGCAGGATTGAAATGTGGC	
SOD2-F	TGCTCCTAAAGCGGTGGTTC	195
SOD2-R	GGGTTGAAATGAGGGCCAGT	
SOD3-F	GAAGCCGCACTAGTAGCCAT	191
SOD3-R	ACGACAGTGAGTGGTTTGGC	
SOD4-F	CGGAGGTCATATCAACCACTC	190
SOD4-R	CAGACCAAGCCACACCCAT	
SOD5-F	ATGTAGGTCTGGTGGTGCTTC	195

SOD5-R	AGAGAAGTGTGTGGATGGCTC	
SOD6-F	CTTTGCTCTTTCCTCACTCT	247
SOD6-R	ACAACCCTCGCATTTCAT	
POD1-F	ACTAAATCTACCACCACCC	172
POD1-R	TGTATCGCCCTTTCCAG	
POD2-F	AGCATAAAAGAGCATCCCTC	179
POD2-R	CACATCCAGACCCAGAAATT	
POD3-F	TTCATTGCGAGGGTTTG	248
POD3-R	TGCTTGAGAAGGCAGATAG	
POD4-F	TACTGGACGCAAAGATGG	244
POD4-R	AGGAAGGAAAGCGGAAT	
POD5-F	TGAGGTGAATGGGAAGAATG	154
POD5-R	AGGTGATGTGGTTGGTGC	
POD6-F	CAAGTCGGCAGTTGAGAAT	227
POD6-R	TGGAAAGGCCCACATTAC	
POD7-F	ACATCTTAGCCGTTGTTGC	182
POD7-R	GGCAGTGAGACCTTTGTTT	
POD8-F	TGTCAACAATTCTGGGCTAC	191
POD8-R	CCACCCTACAGTTCTTCCT	
